# Latest Developments in Industrial Hybrid Machine Tools that Combine Additive and Subtractive Operations

**DOI:** 10.3390/ma11122583

**Published:** 2018-12-18

**Authors:** Magdalena Cortina, Jon Iñaki Arrizubieta, Jose Exequiel Ruiz, Eneko Ukar, Aitzol Lamikiz

**Affiliations:** Department of Mechanical Engineering, University of the Basque Country UPV/EHU, Plaza Torres Quevedo 1, 48013 Bilbao, Spain; joninaki.arrizubieta@ehu.eus (J.I.A.); joseexequiel.ruiz@ehu.eus (J.E.R.); eneko.ukar@ehu.eus (E.U.); aitzol.lamikiz@ehu.eus (A.L.)

**Keywords:** hybrid machines, hybrid manufacturing, additive manufacturing, subtractive manufacturing, Directed Energy Deposition, Powder Bed Fusion

## Abstract

Hybrid machine tools combining additive and subtractive processes have arisen as a solution to increasing manufacture requirements, boosting the potentials of both technologies, while compensating and minimizing their limitations. Nevertheless, the idea of hybrid machines is relatively new and there is a notable lack of knowledge about the implications arisen from their in-practice use. Therefore, the main goal of the present paper is to fill the existing gap, giving an insight into the current advancements and pending tasks of hybrid machines both from an academic and industrial perspective. To that end, the technical-economical potentials and challenges emerging from their use are identified and critically discussed. In addition, the current situation and future perspectives of hybrid machines from the point of view of process planning, monitoring, and inspection are analyzed. On the one hand, it is found that hybrid machines enable a more efficient use of the resources available, as well as the production of previously unattainable complex parts. On the other hand, it is concluded that there are still some technological challenges derived from the interaction of additive and subtractive processes to be overcome (e.g., process planning, decision planning, use of cutting fluids, and need for a post-processing) before a full implantation of hybrid machines is fulfilled.

## 1. Introduction

Manufacturing industries demand efficient processes that provide a reduction in manufacturing costs and required time in order to gain competitiveness while meeting increasing quality standards. Thus, hybrid-manufacturing systems are becoming an industrial solution for the manufacture and repair of high-complexity parts aimed at various sectors [1]. The objective of developing hybrid processes is to enhance their individual advantages while minimizing their limitations [2]. Therefore, they enable one to manufacture components that are not cost-effective or even impossible to manufacture by a single process [3].

The concept of hybrid machines that combine various processes is not new. In 2011, Nassehi proposed a technology-based classification for the different processes that could be integrated into a hybrid machine [4]. These processes are joining, dividing, subtractive, transformative and additive. Nevertheless, due to their irruption, the present article focuses on hybrid processes that combine subtractive and additive technologies.

In view of the market possibilities ahead, many machine tool builders have opted for this solution and started to develop different hybrid machines that combine additive and subtractive operations. Despite the numerous benefits of additive manufacturing, the resulting parts usually require additional machining operations, regardless of the additive approach [5]. This way, hybrid machines have allowed overcoming the main drawbacks associated with additive manufacturing, such as low accuracy and high surface roughness [6]. The combination of both technologies in a single machine is therefore advantageous, as it enables one to build ready-to-use products with an all-in-one hybrid machine [6,7], which maximizes the strong points of each technology [8]. This way, complex components that are originally not possible to be machined due to accessibility constraints are now approachable [9].

**Figure 1:**
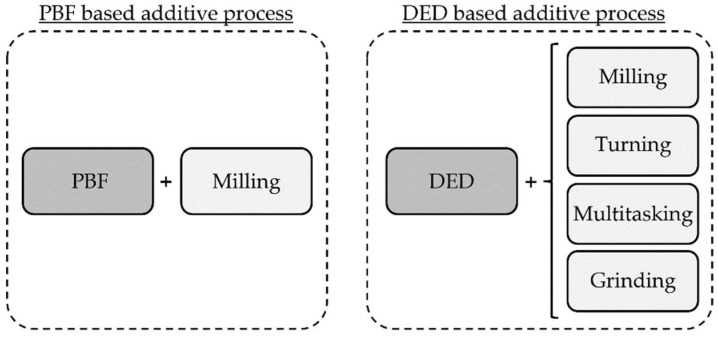
Different process combinations in existing hybrid machines.

The ISO/ASTM 52900 International Standard Terminology for Additive Manufacturing Technologies defines additive manufacturing as the “process of joining materials to make parts from 3D model data, usually layer upon layer, as opposed to subtractive manufacturing and formative manufacturing methodologies” [10]. Among the different metal additive manufacturing technologies available, the industry has predominantly opted for Powder Bed Fusion (PBF) and Directed Energy Deposition (DED) processes [8]. Almost any weldable metal can be processed with any of these two techniques. Nevertheless, the vast majority of hybrid systems integrate Laser Metal Deposition (LMD), which is a DED technology that is faster than Selective Laser Melting (SLM), a PBF process, and does not need any process chamber nor supporting structures [11,12]. For instance, in LMD, typical deposition rate values of 5–30 g·min^−1^ are obtained, whereas the SLM process presents typical values of 2–3 g·min^−1^ [13]. In addition, this approach is adaptable to existing conventional machine tools. Hence, hybrid machines give rise to new opportunities in the manufacturing of high-added-value parts, enabling the high-efficiency production of near-net-shape geometries, as well as the repair and coating of existing components [14]. Besides, the capability to switch between laser and machining operations during the manufacturing process enables finishing by machining regions that are not reachable once the part is finished. In Figure 1, the main additive and subtractive process combinations are shown. They are divided into two groups according to the additive approach they are based on. It is worth mentioning that while PBF-based processes are mainly directed to produce complex whole parts, DED processes are more focused on the generation of coatings. That is why the latter can be combined with a wider range of subtractive processes.

The combination of additive and subtractive processes in a single hybrid machine is especially well-suited for the manufacture of low machinability materials, such as heat resistant alloys and high hardness materials, which are widely used in various industries, including aerospace & defense, automotive, medical, and oil & gas, among others [11]. In fact, this hybrid manufacturing approach has already been used for remanufacturing existing high-added-value components, such as turbine blades [15], integrally bladed rotors [16], gas turbine burner tips [17], or dies & molds [18]. Nevertheless, the full integration of both processes is a complex task that must still overcome many difficulties.

Both laser-based additive processes and machining processes need to overcome challenges on their own in order to improve their performance and enhance the quality of the manufactured parts. For instance, there is a need to study and reduce the environmental impact of the machining processes in order to adapt the technology to the current society requirements [19,20,21]. Besides, there is still work to do in the field of tool path optimization when complex geometries are to be generated, especially for five-axis machining [22,23,24]. The compensation of the volumetric errors generated during the material removal, particularly when big parts are to be manufactured, is also a topic of research [25,26], as well as the analysis of the influence of the vibrations generated during the machining on the resulting surface accuracy [27]. Similarly, the main research areas within the laser-based additive processes are the development of control and monitoring systems that enable the enhancement of the process stability [28,29,30,31,32,33]. In addition, experimental-based [34,35,36] and model-based [37,38] inspection systems aimed at the detection of defects generated during the additive process are being developed. Nevertheless, very little work that focuses on the challenges and benefits that the combination of both technologies involves is found. For instance, authors like Oyeyola et al. have started studying the machinability of DED manufactured parts [39]. However, there is no process combination and the different operations are performed in separate machines.

The present work aims to provide insight into the latest developments in industrial hybrid machine tools combining additive and subtractive operations. To that end, authors have performed a global search in which not only scientific papers have been considered, but also other sources (e.g., industrial magazines, websites, or assistance to machine tool fairs) that cannot be found on scientific databases on the internet. This way, conclusions regarding both industry and academia are extracted. The basis of metal additive manufacturing processes is explained in Section 2, covering both technologies, DED and PBF, most widely used in the industry. In Section 3, the usual configurations of hybrid machines are addressed in terms of kinematics, kind of nozzles and strategies they involve, as well as other distinctive features. Then, in Section 4 and Section 5, issues, such as the potentials and limitations of hybrid manufacturing are dealt with, as well as its presence in the international market from an industrial point of view. Finally, challenges and opportunities in the fields of process planning, monitoring, and inspection, and CAM software development are identified in Section 6, concluding with the global outlook of this manufacturing approach drawn in Section 7.

## 2. Basis of the Metal Additive Processes 

Although different technologies can be distinguished, most hybrid machines use DED as the additive approach. This decision is grounded on the higher deposition rate of the DED processes compared with PBF, together with its capability to add material over a freeform surface.

### 2.1. Fundamentals of the DED Process

The ISO/ASTM 52900 International Standard Terminology for Additive Manufacturing Technologies defines DED processes as “additive manufacturing processes in which focused thermal energy (e.g., laser, electron beam, or plasma arc) is used to fuse materials by melting as they are being deposited” [10]. Among the different DED processes, one of the most broadly used is the Laser Metal Deposition (LMD).

**Figure 2:**
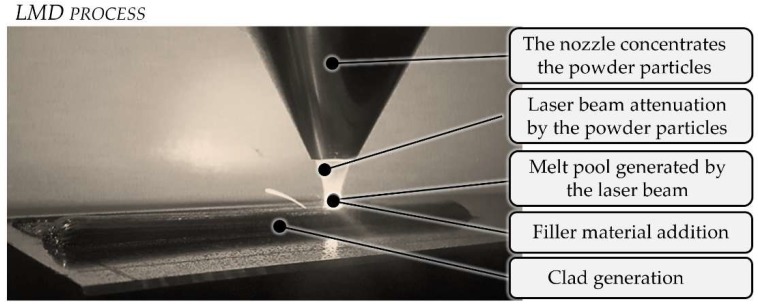
Basis of the Laser Metal Deposition (LMD) process.

Laser Metal Deposition or LMD is probably the most common DED process and it is usually applied to build up fully dense coatings and functional metal parts [40]. In this process, the laser beam generates a melt pool on the surface of the substrate; see Figure 2, while filler material is injected simultaneously through a nozzle, which is also responsible for generating a protective atmosphere that avoids material oxidation [14]. Filler material, supplied in the form of powder or wire [41], is molten by the laser beam and bonded to the substrate, forming clads and subsequent layers until the required geometry is obtained [42]. 

The total amount of energy introduced by the LMD technology into the substrate material is very low when compared to other conventional metal joining techniques, such as arc welding or plasma spraying, which leads to minimum distortion of the workpiece [43]. This creates a fine microstructure, with low levels of dilution between layers and low distortion [18]. All these characteristics give rise to final parts with good mechanical properties and minimal imperfections.

A wide variety of materials common in several industries have been proven adequate for processing via LMD until now and several authors have worked on the experimental determination of the optimal process parameters for the different materials. Some of them are tool steels [44], stainless steels [45], nickel alloys [46], titanium alloys [47], and copper alloys [48]. In addition, LMD is also suitable for adapting the material properties of certain regions of the part to its final requirements and processing Functionally Graded Materials [49,50]. Besides, LMD enables to produce near-net-shape parts, what reduces the material wastage and results in a cleaner and more environmentally friendly process [51]. For instance, typical buy-to-fly material ratios of 4:1 (input material to final component) are common in traditional 5-axis milling processes, with some components having a ratio as high as 20:1 [52]. Nevertheless, LMD is capable of improving these buy-to-fly ratios up to values below 1.5:1 [53]. 

This additive manufacturing process is proved to be effective in the remanufacturing, coating, and repair of existing parts [54], as well as to open new possibilities in the design of innovative geometries [55,56]. For instance, LMD is used for the manufacture and refurbishment applications of critical aerospace engine components [15,57], dies and molds [58], and coatings [59], among others.

However, LMD technology also has limiting factors that make the post-processing of the manufactured parts necessary in order to attain the required final properties. Some examples of the process limitations are the relatively low accuracy of the parts manufactured via LMD and the fact that the resultant surface roughness does not usually match the final requirements [8]. In addition, the anisotropic behavior of the material properties and the generation of residual stresses can lead to geometrical distortions and even cracking of the material [60]. Consequently, continuous corrective measurements during the LMD process are necessary in order to manufacture near-net-shape functional parts with close tolerances and acceptable residual stress [61].

When higher deposition rates are to be achieved, the Wire Arc Additive Manufacturing (WAAM) process is gaining a wider acceptance in the industrial manufacturing sector. This process is a wire-based DED technique that uses an arc-based energy source, which melts the substrate, while wire is used as feedstock material [62]. The components manufactured by this technique are built layer by layer, thus being the deposition procedure similar to that of LMD [63]. Nevertheless, WAAM presents some advantages, such as high deposition rates (up to 10 kg·h^−1^), low equipment cost, and high material utilization. However, high levels of heat input are also inherent to this process, thus inducing residual stresses and high distortion in the built components [64]. As a result, part accuracy and surface finish are lower than in other additive approaches, and a more complex post-processing, usually carried out by subtractive operations, is required. The variety of materials processed by WAAM ranges from nickel alloys and steels to titanium or aluminum [65]. Its main application field is the manufacturing of medium-large size parts of medium geometrical complexity with high mechanical requirements, for instance, aircraft structural components [66].

### 2.2. Fundamentals of the PBF Process

**Figure 3:**
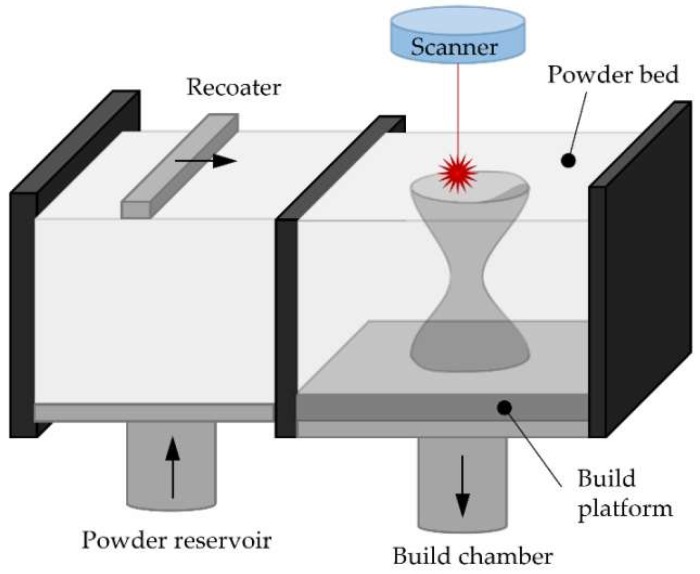
Basis of the Powder Bed Fusion (PBF) processes.

Powder Bed Fusion (PBF) processes are based on the selective melting of determined regions of a pre-deposited powder bed by one or more thermal sources (typically lasers), thus generating a thin layer of material. In order to guarantee a uniform distribution of the powder a levelling system or recoater blade is used. This process is repeated layer by layer until the desired solid is built (see Figure 3). Once the process is completed and the part finished, the metal powder that has not been melted can be sieved and reused [67].

These processes are performed on a build platform inside an enclosed build chamber, which in the case of SLM is filled with inert gas, and often requires support structures in order to improve heat dissipation and keep the part from excessive warping [68]. As a result, both the orientation of the part together with the location of the supports are key factors when setting up a build. Additionally to support structures, the pre-heating of the build platform can also be used for reducing residual stresses. 

Due to the nature of PBF technologies, a broad range of materials, including metals, ceramics, polymers, and composites can be processed, where the main application is the manufacture of full 3D parts with high-complexity geometries. Besides, a good accuracy and resolution are attained for metals. For those reasons, metal PBF processes are becoming increasingly popular for aerospace and biomedical applications, due to their ability to fabricate complex geometries with a wide range of materials and their excellent properties compared to traditional metal manufacturing techniques [69]. Some examples of the application of PBF processes for the production of functional parts are dental and bone implants [70,71], airfoils, or turbine blades with embedded cooling channels [72], thus being able to give service to the aerospace, energy, and medical industries, among others. 

Compared to DED technologies, PBF processes have a relatively slow build rate, but higher complexity and better surface finish can be achieved. However, the size of the build chamber remains as a limitation on the size of the part to be manufactured. In addition, when the PBF manufacturing process is finished, the obtained part must be post processed in order to be separated from the build platform inherent to this technique and remove the supports [73]. 

## 3. Configurations of Hybrid Machines

As stated in the introduction, hybrid-manufacturing systems are becoming an industrial solution for the production of high-complexity parts. In this section, the particularities in terms of configuration as a result of the integration of both additive and subtractive processes in a single platform are addressed.

### 3.1. Kinematic Configurations

**Figure 4:**
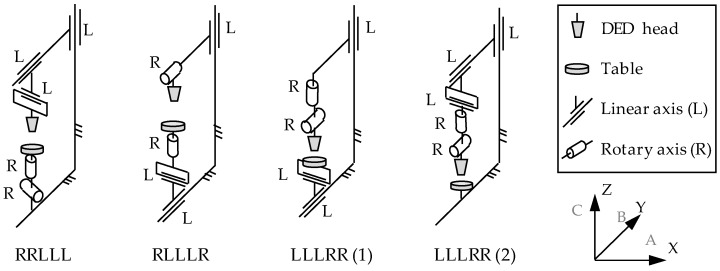
Most common kinematic schemes of hybrid machines.

Aiming at the manufacturing of high-complexity parts, the kinematics of hybrid machines plays an important role in both the accessibility during the process and the resulting accuracy. In Figure 4, the kinematic chains usually employed in hybrid machines are shown. From left to right they are classified according to their suitability for the manufacture of bigger and therefore, heavier parts. Despite 3-axis machines can be also used for hybrid machines, DED processes usually require the deposition of the material normal to the substrate. On the other hand, DED processes are used to be applied to very complex shape parts. Thus, most of the hybrid machines that combine additive and subtractive processes are based on 5-axis machine configurations, on which the present kinematic study is focused.

On the one hand, most hybrid machines are currently based on an RLLLR kinematic chain, where the DED head includes a tilting movement (usually B-axis) and the table includes a rotary table or a universal clamping system (A or C-axis). These types of machines offer a high flexibility together with an elevated stiffness. Moreover, most machine builders have already developed multitasking machines combining turning and milling, where the RLLLR is the most widely used kinematic chain. Therefore, the development of a 5-axis hybrid machine does not imply much design change from the point of view of kinematics, although many changes are required in terms of safety and machine protection.

On the other hand, if the machine is focused on the production of small and complex parts, the use of tilting-table machines, RRLLL kinematic chain, is extended. On the contrary, the biggest parts are manufactured in LLLRR(2) type machines, where the part to be manufactured is fixed and all moving axes are situated in the DED head.

### 3.2. Nozzles and Strategy Restrictions

In order to obtain a stable process, simultaneously to the generation of the melt pool on the surface of the substrate, filler material needs to be directed and injected using a specific nozzle. There are different types of nozzles for powder-based DED processes and based on their geometry and depending on the powder injection system, three nozzle types can be distinguished: off-axis, coaxial discrete, and coaxial continuous [74]. The design of the nozzle is a key factor that has a direct influence on the powder pass distribution at the nozzle exit and therefore determines its efficiency. In addition, the design type also determines the kind of application of the nozzle, restricting the operations in which it is suitable: off-axis nozzles are usually employed for coatings, while coaxial nozzles are used for building 3D parts.

The off-axis nozzles are the simplest and most economical, where a single powder stream is fed laterally into the laser beam. However, as their powder feed is dependent of the direction, their use is restricted to a unidirectional deposition strategy, mainly to the coating of rotary parts, being unable to build 3D parts. Therefore, their use in hybrid machines is residual.

**Figure 5:**
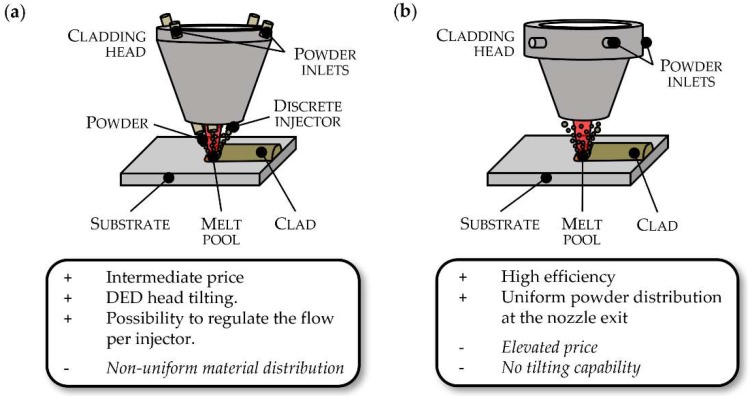
Different nozzles used in powder-based DED: (**a**) Discrete and (**b**) Continuous coaxial nozzles. Reproduced with permission from [76], Elsevier, 2017.

As an evolution of the off-axis, coaxial discrete nozzles have been developed (see Figure 5a), which enable multidirectional deposition at an intermediate price. Their working principle is based on a number of discrete injectors that are positioned around the rotary axis of the nozzle and powder particles are fed coaxially to the laser beam. Depending on the design, three or four injectors may be positioned, whose powder flow can be regulated independently. However, due to its working principle, the powder distribution obtained is not uniform. The configuration of the coaxial discrete nozzles enables the tilting of the DED head up to 180° and therefore 5-axis deposition [75].

Lastly, coaxial continuous nozzles have also arisen (see Figure 5b), generating an axisymmetric flow of powder at the nozzle exit that encloses the laser beam, thus being able to build 3D parts. In this case, a higher efficiency of the nozzle can be achieved, as the diameter of the powder stream can be adapted to the size of the laser beam on the workpiece [77]. In addition, the powder distribution is ensured to be uniform and homogeneous at the nozzle exit. However, due to their complexity, these kinds of nozzles are expensive. Besides, their tilting is restricted due to the effect of gravity on the powder cone. Experiments show that this system can work satisfactorily with a maximum tilt angle of 20° [75].

### 3.3. Other Features 

The introduction of powder particles in the working envelope of the hybrid machine in order to perform additive operations forces to make some considerations in order to preserve the integrity of the moving elements. Thus, machine tool builders have started to take measures to address this issue. For instance, some manufacturers (e.g., Mitsui Seiki, Okuma) have incorporated the same features as in their machining centers aimed at the machining of graphite, during which graphite dust is generated. That is, the machines include a fully enclosed guard that completely seals powder particles inside and then extracts them by means of an exhaust system. In addition, kinematic protections are also implemented in order to protect the moving elements from metal powder [78,79]. Furthermore, another kind of safeguard to be taken is that against sparks due to static discharges.

The combination of additive and subtractive technologies also has certain implications to be considered so that one process does not affect the other negatively. One example of it is the cutting fluid–laser tandem. In order to remove the excessive cutting fluid from the part to be processed additively, some manufacturers are opting for blowing it off the part [80]. However, this is not an advisable practice when metal powder is present, as it poses fire and explosion risks [81].

## 4. Study of the Capabilities of Hybrid Machines

The combination of additive and subtractive technologies in a single hybrid machine brings unquestionable advantages for the production of complex parts. However, not only are there positive aspects. Hence, in this section, a critical analysis of the capabilities of hybrid machines is performed, including their potentials, as well as the challenges still to be faced.

### 4.1. Potentials of Hybrid Machines

**Figure 6:**
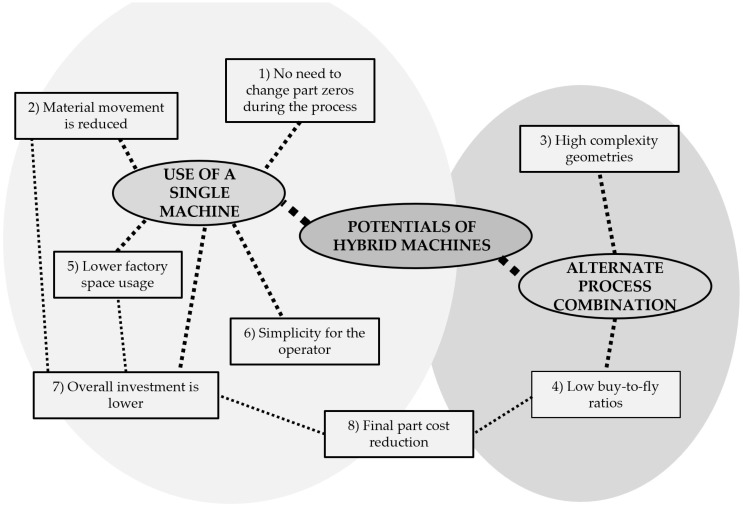
Potentials of the hybrid machines.

The development of hybrid machines has enabled us to unite the advantages of multiple processes using a single machine for the whole manufacturing of metallic parts with the subsequent benefits that it brings (pre and post process operations that require other machines or working stations might still be necessary: heat treatments, painting operations, etc.). The most relevant strong points derived from the use of hybrid machines are presented in Figure 6 and expanded upon in the subsequent list.

1. There is no need to change part zeros during the manufacturing process. A single setup is used for both additive and subtractive operations [82]. Therefore, the part-positioning error is minimized, which results in a higher final accuracy. Furthermore, as there is only the need for a single zero-setup per part or set of parts, non-productive time due to zero making is reduced to minimum values.
2. Material movement inside the factory is reduced. Hybrid machines enable one to manufacture whole parts in the same machine, without the necessity to move the part to other machines for finishing operations [83]. Hence, intermediate warehouses are eliminated from the factory, which results in a better use of the available space. Besides, as the movement of the material is minimized, on the one hand, the workload of the equipment for material handling is reduced and, on the other hand, the chances for collisions and accidents are lowered, which results in an increase of the employees´ safety.
3. Manufacture of higher complexity geometries. The hybrid machine can switch between additive and machining operations seamlessly during the manufacturing of a single part [84]. Therefore, it is feasible to machine areas that are no longer accessible once the part is finished. This results in a higher freedom and flexibility when designing the optimum geometry of the part. Besides, this point is directly related with the previously introduced issues 1 and 2, because when the manufacturing process switches between additive and subtractive operations, there is no material movement inside the factory, nor precision lost due to zero changes between different manufacturing platforms.
4. Low buy-to-fly ratios. The possibility to generate near-net-shape components using additive manufacturing results in a reduction of material waste, as well as the economic costs related to the material recycle and waste treatment. Buy-to-fly ratios as low as 1.5:1 are achieved thanks to hybrid machines [52]. Consequently, the ecological footprint resulting from the process is reduced. Combining additive and subtractive operations enables to take advantage of the potential of both processes and therefore, material-efficiencies up to 97% can be achieved [85,86].
5. Lower factory space is used. Thanks to the use of hybrid machines, additive and subtractive operations can be carried out within the same machine, the number of required machines for the manufacture of a certain part is reduced. Therefore, occupied space in the workshop is also reduced.
6. Simplicity for the operator. The integration of both processes in a single machine under a unique interface means that the operator must only deal with one working station, which simplifies training as well as daily work.
7. The overall investment is lower. Hybrid machines are more expensive than simple additive or milling machines. However, the integration of both technologies in a hybrid machine involves sharing common elements (e.g., guiding systems, machine tool structure, CNC control, user interface). Hence, the total investment required for the acquisition of a hybrid platform is considerably lower than buying two separate machines.
8. Reduction of the costs of the final part. Additive manufacturing enables realization of high-performance coatings over ordinary or “cheap” materials, thus achieving a final part with enhanced properties, but at a cheaper cost.

### 4.2. Challenges of Hybrid Machines

**Figure 7:**
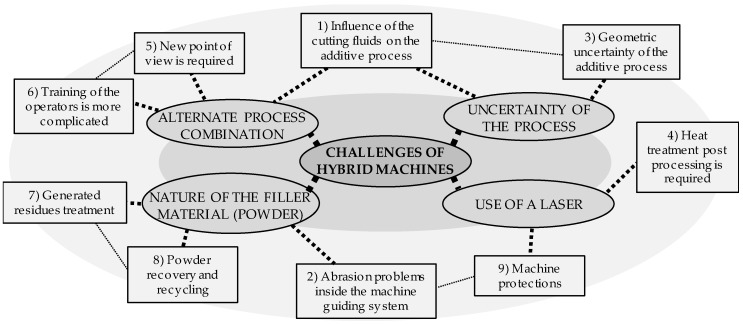
Challenges of hybrid machines.

Despite the numerous potentials of the hybrid machines, many issues still need to be solved when combining additive and subtractive processes. In Figure 7, the most relevant challenges to be faced before their complete implementation are shown and their connections are highlighted.

1. Influence of the cutting fluids on the additive process. The remains of the cutting fluids from the machining process can influence the subsequent additive process [87]. This issue has a double effect. On the one hand, powder particles mix with the fluid and generate moisture that directly influences the laser beam absorptivity and the dilution of the filler material within the substrate. On the other hand, the cutting fluid vaporizes during the additive process and results in porosity increase, as well as possible damage to the optical systems due to the contamination of the lenses [88,89].
  As a result, ensuring the cleanliness of the substrate is of great importance in order to avoid internal defects (e.g., porosity, cracks), guarantee a good bonding between the deposited and base materials, and therefore perform a high-quality additive operation [90]. This means that, as far as hybrid machines are concerned, an intermediate cleaning stage between machining and the subsequent additive operation is necessary. However, there is no agreement neither in the industry nor in academia about how to proceed with regard to this issue and only a few studies can be found [87,88].
  Problems with cutting fluids are even more critical when PBF processes and milling are combined. In this case, no cutting fluids can be used in the subtractive operation, as their mixture with the powder bed would be detrimental for the whole process, which results in lower feed rate, lower plunging depths, higher tool wear, etc. when machining.
2. Abrasion problems inside the guiding system of the machine. Hybrid machines require a special protection that preserves the guiding system from the powder used in the additive operation [78,79]. The powder particles used in DED and PBF processes have diameters ranging between 45–150 µm and 10–40 µm, respectively. Therefore, if the machine is not properly sealed, powder particles might intrude and interfere with the smooth movement of the guiding system, as well as with the encoders used for determining the position in the machine.
3. Geometric uncertainty of the additive process. Due to the uncertainty that additive manufacturing suffers as a consequence of the state of the art and lack of maturity of the technology, the additive stage is the weakest link within hybrid machines. For example, it is well known that additive manufacturing can produce complex internal features, but there is a lack of knowledge regarding how those features should be inspected [91].
  Regarding the accuracy of the additive process, especially in DED operations, it is lower than that of machining. In Table 1, a comparison between PBF and DED technologies’ dimensional accuracy and surface roughness is presented [92]. As it can be seen, DED is a less accurate process than PBF. However, in both cases, a post-processing stage is necessary depending on the final requirements of the part.

**Table 1:** Comparison between PBF and DED technologies.

Technology	Dimensional Accuracy (mm)	Surface Roughness (µm)	Ref.
PBF	±0.05	9–16	[93,94]
DED	±0.13	≈40	[95,96]

The material deposition rate in the additive process is extremely sensitive to the feed rate of the machine, the volume of the substrate, the geometry of the region where the material is being deposited, the surface finish, etc., which may generate differences between the originally designed and finally manufactured part [97]. Besides, the internal stresses generated during the additive stage due to the thermal nature of the process may generate considerable geometrical distortions. Therefore, in the subsequent machining operation, the tool may encounter material over-accumulations and different geometries from those expected, which may lead to the breakage of the cutting tool.
4. The requirement of a post-processing heat treatment. During the additive process, the material is subjected to heating and cooling thermal cycles, which leads to the generation of residual stresses that might be released during the subsequent machining operations. This results in distortions of the part geometry and hence the machining tool may encounter different plunging depths to those programmed [98]. Besides, the mechanical properties of the deposited material, for instance ductility, are very sensitive to the presence of internal defects and porosity [99].
  In order to reduce internal stresses and solve these issues, additively manufactured parts usually require a post-processing heat treatment [100]. For example, Kobryn and Semiating studied the influence of a post-processing stage on a Ti-6Al4V part produced by Laser Engineered Net Shaping (LENS) and concluded that a Hot Isostatic Pressing (HIP) operation can increase ductility from 0.8% to values of almost 12% [101]. Besides, authors like Åsberg et al. studied the influence of HIP on the yield strength of an AISI H13 tool steel and a 30% improvement was obtained with regard to the as-deposited material, reaching an average value of 1502 MPa [102].
  Therefore, in case the hybrid machine is not prepared for providing the required heat treatment, material movement to an external furnace is mandatory, which eliminates one of the main advantages of using a hybrid machine.
5. Necessity of a paradigm shift. Design engineers need to learn not only each process independently (additive and subtractive), but also the possibilities that have arisen thanks to their combination, which may result in a change of the whole concept of designing parts. For instance, Salonitis and Zarban proposed a methodology for redesigning the geometry of a part to be additively manufactured based on a Multi-Criteria Decision Analysis (MCDA) for assisting in decision-making [103]. However, there is no standard methodology or process planning aimed at hybrid machines.
  Besides, Hällgren et al. stated that additive manufacturing could be approached from two points of view: design-driven and process-driven [104]. The first one focuses on improving the geometry of the manufactured part, but without considering the optimization of the manufacturing process itself, whereas the second one works the other way. However, if satisfying results are to be obtained and the full potential of hybrid manufacturing is to be exploited, a new point of view that combines both ideas is required.
6. Training of operators is more complicated. Operators require a wider background knowledge to master in both processes; hence, this is directly translated into a longer training period. Moreover, due to the extensive freedom of additive manufacturing, hybrid machines usually require the use of computational frameworks in order to optimize both processes [105]. Besides, operators must be trained in safety issues, especially with regard to the powder handling and laser operating [106].
7. Generated residues treatment. Thanks to the capability of the additive manufacturing to generate near-net-shape geometries, hybrid machines reduce the amount of generated waste material by as much as 90% [107]. However, their treatment may be far more complicated, from a logistical point of view, and economically expensive. Special attention must be paid to powder handling, recycling of the liquid wastes (e.g., lubrication oils used for the movement of the axes, cutting fluids employed in machining). Depending on the composition of the used powder particles, the residues are extremely hazardous to human health. Especially, powder with high content of nickel or cobalt are carcinogenic to our health. All this obliges the company to install special protective measures, as well as a proper protocol for treating the residues [108]. Moreover, despite the latest advances, there is a lack of knowledge and studies related to the toxicity and harmful effects related to the powder particles [109]
8. Powder recovery and recycling. In laser metal additive processes, only a fraction of the fed powder is melted by the laser and added to the substrate, whereas the rest is lost. Industrial powder based DED systems have an efficiency ranging between 5 and 70% [110], while in wire-based DED and PBF much higher efficiencies are obtained, reaching values of almost 100%. However, this powder has interacted with the laser beam and consequently, the shape and composition of the particles may have changed, which is detrimental for its reutilization. Carroll et al. concluded that the reused powder reduces the hardness of the deposited material and increases the surface roughness [110]. Besides, it may have contacted with substances, such as oil, dust, other composition powders, etc., negatively affecting the process. In order to highlight the discrepancies between the different authors and the existing uncertainty in this field, some authors conclude that Inconel 718 DED powder can be reused twice [111], whereas others increase this number up to 10 reuses without major changes in the results for the Inconel 625 DED [110].
9. Machine protections. Besides protecting the machine operator from collisions, the guarding of the hybrid machines needs to be capable of retaining the high-intensity light generated by the laser inside the machine and to withstand the heat generated during the additive process. Reflections of the laser beam when highly reflective materials are being processed (e.g., aluminum, copper) may result in the melting of specific areas of the guarding or other sensitive elements and, therefore, proper protection must be arranged.

## 5. Latest Developments from an Industrial Perspective

Despite the potential of additive manufacturing, its application also has some limitations, such as accessing difficult to reach areas inside complex parts. This issue is one of the main reasons why many machine tool builders have opted for offering hybrid systems that combine additive and subtractive processes. 

In this section, the latest hybrid machines developed by the market-leading machine tool builders are detailed with the aim of providing readers a broad vision of the market situation and the latest launches. They are grouped into two categories, depending on the additive approach, DED or PBF, employed.

### 5.1. DED-based Hybrid Machines

Many machine tool builders are successfully operating in the market of additive manufacturing using both DED and PBF technologies. However, when it comes to hybrid systems, the availability of machines based on DED technology is noticeably wider than those integrating PBF processes. Some of the underlying reasons for this situation are the much higher deposition rate that DED offers, as well as the possibility of adding material on existing parts. Moreover, thanks to the feasibility of depositing material while the five-axis of the machine are simultaneously interpolated, complex geometries can be built without any support structure. 

One of the companies that has strongly invested in hybrid machines integrating DED processes rather than PBF is the German-Japanese DMG MORI, who aims to gain access to the aerospace, energy, and die and mold industries with the help of LASERTEC 3D hybrid series. To that end, they already provide two different hybrid solutions: *LASERTEC 65 3D hybrid* and *LASERTEC 4300 3D hybrid*, launched in 2014 and 2016, respectively. The first one, based on an RRLLL kinematic chain, includes a tilting table and combines the flexibility of LMD with the precision of five-axis milling [112]. Alternatively, the second one includes a B-axis tilting movement in the head and an RLLLR kinematic chain, integrating six-axis LMD and five-axis turning/milling operations. In addition, this machine is equipped with up to five nozzles of different sizes and an automatic laser head changer [113]. Both machines are provided with process monitoring and control devices, such as real-time temperature and melt pool size measurement that enables automatic laser power regulation.

**Figure 8:**
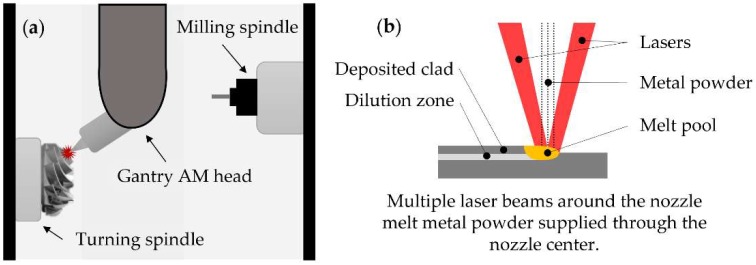
(**a**) Gantry AM head used in the *INTEGREX i-200S*; (**b**) Multi-Laser Deposition process. [118].

Based on its extensive expertise in building multitasking machine centers, the Japanese Mazak has also made inroads into the hybrid machine’s market with five DED-based hybrid machines. The *INTEGREX i-400AM* [114], launched in 2014, combines 5-axis machining and LMD under a RLLLR kinematic configuration. The machine can switch between two different laser processing heads that are loaded into the milling spindle by a standard tool changer, aiming high speed or high accuracy deposition and enabling the adjustment of the deposited clad size depending on the process requirements and employed material. Two years later, in 2016, the *VC-500 AM* hybrid multitasking machine was presented [115]. This machine, which is only available in the US market, features 5-axis milling and LMD additive technology. Alike the *INTEGREX i-400AM*, the *VC-500 AM* is based on an RRLLL kinematic chain, which includes a tilting-table, A and C axis, and the translation movements are situated in the LMD head. Besides, the machine is provided with the Mazak *MAZATROL SmoothX* CNC technology [116], which eases the generation of programs for manufacturing of complex parts. In the same year, 2016, the *INTEGREX i-200S AM* was introduced at JIMTOF 2016 [117]. This hybrid machine tool uses an RLLLR kinematic chain, including two turning spindles, a milling spindle, and a Gantry AM head (Figure 8a), and integrates Multi-Laser Metal Deposition with milling/turning. Multi-Laser Metal Deposition is a process in which multiple laser beams are used to melt metal powder fed through the center of a laser head, attaining a stable metal powder flow even when tilting the laser head, and provides very high accuracy (Figure 8b). The evolution of this machine, the *INTEGREX i-300S AM*, was also launched and presented at the Machine Tool World Exposition (EMO) 2017.

The *VARIAXIS j-600/5X AM* [119], launched in 2016, is based on a vertical 5-axis machining center combined with WAAM. This machine is based on an RRLLL kinematic chain, where a tilting table is used and the additive manufacturing head includes linear movements. 

Another company that has also broken into the hybrid market is the Japanese Okuma, who, since 2016, has included the LASER EX series within its portfolio. This machine series, developed in collaboration with TRUMPF, combine subtractive and additive functionalities, hardening, and coating in a single platform. On the one hand, the *MU-V LASER EX* [120] machines are 5-axis vertical machining centers provided with laser processing capabilities. All MU-V machines are based on an RRLLL kinematic chain, where the tilting table includes the X linear axis and the Y and Z are included in the DED/milling head. On the other hand, the *MULTUS U LASER EX* [121] series are based on a 5-axis horizontal multitasking machine. However, unlike the vertical centers, these horizontal centers include an RLLLR kinematic chain, together with a B-axis tilting head.

The worldwide manufacturer WFL, who concentrates uniquely on the production of multifunctional complete machining centers, has integrated laser-based additive technologies into a MILLTURN multi-task machining center. This way, fully integrated laser solutions, such as laser cladding, laser welding, and laser hardening, are possible on the *M80 MILLTURN* [122], which is also provided with turning, boring, and milling functionalities. The machine’s kinematic chain is based on an RLLLR configuration, where the DED/milling head includes a B-axis tilting movement.

The Basque machine tool builder Ibarmia has also decided to start incorporating laser cladding functionalities in its 5-axis multitasking machines, giving rise to the ADD+PROCESS series and the *ZVH 45/L1600 ADD+PROCESS* machine. This machine is based on a moving column architecture with a B-axis tilting head, RLLLR kinematic chain, and combines DED with precision milling and turning. Besides, the laser spot size can be modified in order to attain high productivity or fine geometries by DED [123].

Mitsui Seiki also launched in 2016 the hybrid machine *Vertex 55X-H*, which integrates a spindle-adapted laser DED system into a traditional high precision machine tool [124]. The machine is based on an RRLLL kinematic chain with a tilting table and a gantry-type structure. Especially notable are the measures this company has taken in order to face the problems arisen due to the combination of additive and subtractive operations. On the one hand, the machine includes an air blow-off operation that removes much of the volume of coolant still on the part, followed by a laser cleaning stage (with the laser defocused) prior to the additive operation [79]. On the other hand, in order to avoid problems in the bearings and axis guiding systems, the machine has guarding and other kinematic protections that the company has adapted from other milling machines that are especially designed for working with graphite.

Despite traditionally integrating machining operations, the hybrid manufacturing and hybrid machine tool concept are also open to other alternatives. One example of this is the *millGrind* hybrid machine developed by Elb-Schliff WZM GmbH, which combines DED and grinding technologies, offering, according to the company, 0.1 µm accuracy [125].

### 5.2. PBF-based Hybrid Machines

Despite most of the manufacturers opting for DED-based hybrid machines, there are also PBF-based alternatives that are worth mentioning. For instance, Sodick, a Japanese company focused on the manufacturing of Electrical Discharge Machining (EDM) and high speed milling center machines, has developed the new OPM series, comprised by *OPM250L* [126] and *OPM350L* [127] which perform both SLM and high-speed milling. Each layer is milled as soon as it is built so that a high-quality accuracy and precision are attained, even on cavities or inner features that are not reachable once the part is finished. Therefore, these hybrid machines are specially designed for the manufacture of mold cooling channels.

**Figure 9:**
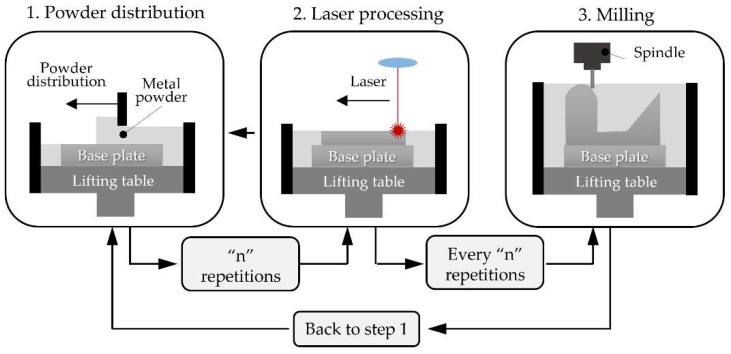
Additive and subtractive operation combination on which the Matsuura hybrid machines are based [131].

Meanwhile, the high-speed machining centers manufacturer Matsuura has developed, since 2003, Metal Laser Sintering Hybrid Milling Machines under the name LUMEX Series [128]. This series consists of two different machines that differ in the size of their working area, *Lumex Avance 25* [129] and *Lumex Avance 60* [130]. In both models, an Ytterbium fiber laser is installed and the milling operation is performed by default after every 10 layers are processed, as seen in Figure 9.

Finally, yet importantly, GF Machining Solutions has collaborated with different additive manufacturers and now is developing joint solutions with the US company 3D Systems in order to develop the *DMP Factory 500* [132,133]. The machine is grounded on a Direct Metal Printing-based additive manufacturing platform, in which a *System3R tooling system* has been incorporated [134]. This tooling system enables one to switch the part between the different additive and subtractive machines.

Therefore, the proposal of GF and 3D Systems is not a hybrid machine itself, but a special tooling system that claims to reduce the setup time to a few minutes and to guarantee precision and accuracy during the entire manufacturing process chain. This way, they compare their process to that performed in a hybrid machine.

## 6. Current Situation and Future Perspectives

The development of hybrid machines combining additive and subtractive processes opens doors to a new concept in terms of both design and manufacturing, enabling the construction of new components previously beyond reach. Nevertheless, the consideration of both technologies in holistic terms is a pressing need in order to perform their full integration within a single platform as a comprehensive manufacturing approach. This necessity gives rise to a new vision of the process, conscious of the requirements and restrictions of each technology and leveraging their potentials. In order to grasp its importance, the most relevant advances made in this direction are addressed in terms of process planning, monitoring and inspection, and CAM (Computer-Aided Manufacturing) software developments.

### 6.1. Process Planning

**Figure 10:**
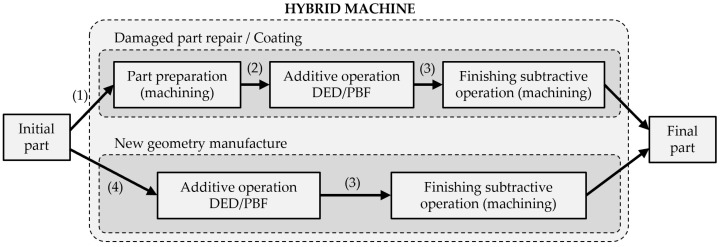
Process interactions within a hybrid machine.

An integral view of hybrid processes combining additive and subtractive operations is required in order to optimize the interaction of both technologies during the production of a component. Hybrid manufacturing is especially interesting for the manufacture and repair of high-added-value parts. Depending on whether a new geometry is to be manufactured or a damaged existing part needs to be repaired, a different approach is adopted, as shown in Figure 10. The manufacture of a new geometry starts with the addition of material followed by a finishing operation, usually by machining, so that the requirements of the final part are met. By contrast, the process sequence required in a repair or coating context may involve alternating additive and subtractive operations. In this case, the interaction between both processes becomes especially important.

Nevertheless, some considerations to be taken are represented in the figure. On the one hand, when repairing a component, (1) the initial part needs to be measured and characterized before any operation is performed. Then, (2) an intermediate cleaning stage between the subtractive and additive operations is required in order to remove the cutting fluid remnants from the area to be additively processed. In addition, after performing any additive approach, (3) the resulting component needs to be measured so that the outcome of the additive operation is checked, and the subsequent machining tool paths are defined. On the other hand, when a new geometry is to be built, (4) the initial part or substrate needs to be inspected and subsequently clean in order to ensure a good quality of the deposited material. Similar to the previous case, the dimensional characterization of the additively built-up part (3) is crucial in order to proceed with the finishing operation. Nevertheless, the difference lies in the fact that, for repair/coating of a damaged part, a preparatory machining operation is required before any additive approach, due to the use of cutting fluids. As explained in Section 4.2, cutting fluid remnants on the part negatively affect the additive process. Therefore, this sequence of operations makes the introduction of a cleaning stage inside the hybrid platform a must, thus directly affecting process planning. On the contrary, for the manufacture of a new geometry, the substrate material can be just visually inspected and preliminarily cleaned (e.g., manually) before being introduced into the hybrid machine.

So far, few researchers have focused their efforts on process planning for combined additive and subtractive manufacturing technologies. Ren et al. [18] defined a process planning procedure for repairing dies by DED and machining. For that purpose, the authors identified the following sequence of operations: (1) determining the features to be repaired, (2) machining the damaged features, (3) additive operation to repair the feature, and (4) finishing of the deposited material by machining. Nevertheless, problems that may arise from the geometrical distortions generated during the additive process are not considered. On the other hand, Kerbrat et al. [135] proposed a methodology based on manufacturability indexes for identifying features able to be manufactured by additive processes during the product design stage. Besides, Le et al. [136,137] generated a manufacturing process sequence aimed at reincarnating end of life (EoL) or existing components into final parts with new functionalities. In a first step, the authors identified machining and additive manufacturing features by comparing the CAD (Computer-Aided Design) models of the existing and the objective geometries and considering the restrictions of each technology. Then, the process plan is designed by respecting relationships between features, rules based on manufacturing precedence constraints and tool accessibility. However, process factors, such as the importance of maintaining a constant processing velocity during the DED process and the influence of the coolant used in previous machining stages, are not considered. More recently, Behandish et al. [138] developed an early approach for automatic evaluation of manufacturability and generation of process plans for hybrid manufacturing via computer-aided process planning. Thus, process planning is moving towards combining additive and subtractive operations from a holistic perspective and not only applying machining as a preparatory or finishing operation.

### 6.2. Monitoring and Inspection

The implementation of in situ monitoring and inspection systems enables obtaining immediate information from the manufacturing process and early detection of defects or anomalies. This way, the quality of both the final part and the process is enhanced, while the number of rejections and amount of scrap are reduced. To that end, process parameters need to be controlled instantaneously depending on external variables and many authors have focused their efforts on monitoring the process during the last decade [139,140,141,142].

As far as DED processes are concerned, temperature monitoring is of major importance, as the melt pool temperature is a relevant parameter that affects both the metallurgical quality and geometry accuracy of the manufactured component [143]. In addition, the height of the layers deposited by this technique does not stay constant throughout the process, which makes the subsequent inspection of the produced part crucial. Literature shows that researchers have made efforts in monitoring the size and temperature of the melt pool. For instance, some authors suggest the integration of imaging sensors into the nozzle, aiming to control the width of the melt pool and, consequently, the quality of the deposition [144,145]. Hofman et al. developed a feedback control algorithm that enables one to adjust process parameters in situ in order to control the melt pool size [146] and measure the clad height in real time [33,147]. Furthermore, aiming to control the microstructure of the deposited material, Farshidianfar et al. processed thermal information in real time [148]. Aware of the growing interest aroused by monitoring and control, Siemens is currently looking for cooperation with additive manufacturing OEMs to develop and implement process monitoring into different additive manufacturing processes. [143]. Nevertheless, these monitoring systems are only capable of acting on the process once they have detected a deviation from the set values. Therefore, in order to obtain higher quality parts and avoid waste, a look-ahead monitoring system should be developed, which not only acts on the process variables according to the instantaneous measurements, but that can also predict what will happen in the following steps and act accordingly.

Moreover, due to the relatively low dimensional accuracy of the additive processes and the related uncertainty, in many cases, it is necessary to measure the additively manufactured part before the subtractive stage. For instance, Campbell et al. implemented a 3D visualization algorithm based on AutoCAD software that evaluates the surface roughness, compares the geometry with the theoretical, and detects any potential problematic areas [149]. Similarly, Mandić et al. proposed an ATOS GOM 3D scanner for measuring the external geometry of the additively manufactured part [150], whereas with the aim of obtaining a more accurate measurement of specific areas, Newton et al. proposed using focus variation microscopy for measuring the roughness resulting from the PBF additive process [151]. On the other hand, Townsend et al. employed an X-ray computed tomography system for detecting internal defects [152]. Nevertheless, in order to take maximum advantage of the potentials that hybrid machines offer, the geometric evaluation of the part needs to be performed inside the machine.

Besides, sometimes the geometry of the substrate must be accurately defined before determining the DED strategies for the additive process [153]. In this direction, Liu et al. developed a set of algorithms and numerical methods to generate the most suitable tool paths and enable DED process automation. If the distance between the nozzle and the substrate departs from its optimal value, the powder–laser interaction is altered, resulting in process growth variations and, consequently, reduced deposition quality and geometric inaccuracies [154,155].

In addition, Siemens has also made inroads into part inspection by integrating computer-aided inspection tools in an NX environment. This way, the software enables validation of the quality of printed parts by Coordinate Measuring Machine (CMM) inspection programming together with the visualization and analysis of the data so that both theoretical CAD and real geometries can be easily compared [156].

### 6.3. CAM Software Developments

In metal additive manufacturing, built parts are usually finished by means of subtractive technologies. However, the combination of both processes in a unified software solution allowing a holistic process planning remains as a challenge and thus a matter of research.

As far as the additive operation is concerned and regarding the LMD process, which is the most extended technology in hybrid machines, the main applications are focused on the generation of coatings and the repair of high-added-value components. However, the fact that the manufactured parts may have complex geometries and the LMD process orientation must be kept normal to the substrate requires one to interpolate the five axes of the machine simultaneously in order to obtain the desired part.

The trajectory generation for the subtractive processes is relatively well solved because it is mainly a geometric problem. However, the additive process is more sensitive and the resulting geometry depends on many more factors, such as the size of the part, the duration of the additive operation, and the complexity of the trajectory to be followed.

**Figure 11:**
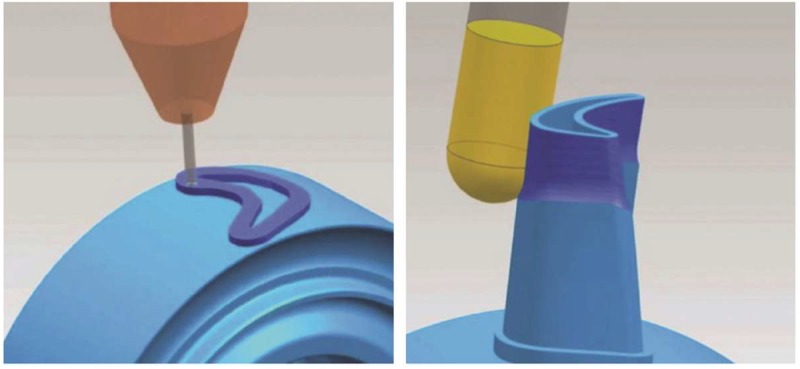
In-process workpiece and verification work for both additive and subtractive modes, by Siemens PLM Software [161], used under CC BY-ND 2.0.

In order to solve this issue, the Fraunhofer Institute for Laser Technology (ILT) has developed a tool called LaCam3D that enables both programmers and end-users to generate tool paths and translate them into the CNC machine code. Furthermore, it allows simulating the process and identification of possible collisions [157]. In addition, some researchers are also working on the development of Application Programming Interfaces (APIs) implemented on commercial software, aiming to offer full solutions for hybrid processes [158]. Siemens is also collaborating with DMG Mori [159] on the development of the PLM software *NX Hybrid Additive Manufacturing*, having both additive and subtractive manufacturing functions in a single software. Although this software is not yet commercially available, further details can be found on their webpage [160]. As can be seen in Figure 11, the in-process workpiece designed in the NX CAD module can undergo both additive and subtractive operation in any order.

This is the first commercial solution that allows CAD/CAM additive operations and was presented in Milan at the EMO in 2015. The developed *NX Hybrid Additive Manufacturing* module is currently specifically configured for the Lasertec 65 3D from DMG Mori and the Siemens Sinumerik 840D CNC control system.

However, the additive process is extremely sensitive to the feed rate of the machine and the thermal field of the substrate and, therefore, the tool paths calculation is not a trivial task as in the case of the machining operations. Thermal simulations may be a solution to the problem, because they enable calculation of the thermal field as well as the clad height, but due to their extremely high computational cost, a combination of CAD/CAM and experimentally-based databases seems to be the best solution.

## 7. Conclusions

There is no doubt that additive manufacturing technologies are changing the paradigm of production. Their combination with subtractive operations helps to overcome the low accuracy, precision, and high roughness usually related to additive manufacturing, hence enabling the production of components that were previously unattainable. Nevertheless, the integration of additive and subtractive operations into a single machine, although the latter is a mature technology, is certainly not without its challenges.

On the one hand, the main benefit of hybrid machines can be outlined as a more efficient use of the available resources, resulting in shortening of the process chains, with subsequent time and economic savings. At the technical level, hybrid machines open the possibility of manufacturing higher-complexity components, thus enabling the construction of more flexible new designs with enhanced characteristics. On the other hand, the vast majority of challenges that hybrid machines must still face come from the additive side and its lack of maturity. In this direction, the industrial sector has already started to make some considerations when building these new machine tools so that issues, such as safety of the user, guarding of the machine or residues treatment, among others, are addressed.

However, and from a technical point of view, there is still work to be done with regard to understanding additive technologies and their interaction with subtractive processes before the full implementation of hybrid machines can be realized. Here, we propose some issues that are currently underexplored and need more attention:

- The prediction of issues, such as clad geometry, porosity, hardness, microstructure, or residual stresses, is highly important in order to build an accurate, high-quality component. For instance, the combination of thermomechanical models with CAD/CAM software is of great importance in order to be able to design adequate building strategies and guarantee there is no excessive deviation between the projected and the actual part. Nevertheless, the use of experimentally obtained databases that correlate process parameters and the obtained clad geometry is gaining wide acceptance due to their lower computational cost and faster solution.
- Process monitoring is of great relevance when ensuring the quality of additively manufactured parts. Besides, they can provide information regarding the process and the probability of defect apparition. Nevertheless, the future of monitoring cannot be limited to controlling the process instantaneously, but also to be capable of predicting defects before they occur and acting accordingly in order to avoid their appearance.
- Alternatives to the application of heat treatment in a furnace, which is commonly used for reducing residual stresses generated in the additive operation, are also required. The investigation of laser-based heat treatments inside the hybrid machine in combination with further heat treatment stages once the component is out of it could help overcome the setback of eliminating the furnace-based intermediate treatment stage.
- In addition, there is still a lack of agreement on some aspects, such as the best means to eliminate metal powder or cutting fluid from the working environment so that their mixture does not affect the additive operation. In this regard and based on experience, the authors believe that the best way to proceed would be a combination of powder blowing and laser cleaning prior to the additive process. However, the literature shows that different materials react unequally, in terms of porosity, cracking, and microstructural, to the presence of cutting fluid remnants during deposition. Thus, further research on either advancing towards dry machining or analyzing a wider range of materials is needed in order to understand and/or avoid this kind of interaction.
- Decision and process planning for hybrid solutions are major outstanding issues, where substantial progress needs to be made. The main challenge is now the development of a software tool that allows the design of an optimized process plan comprised of enhanced additive and subtractive operations that can be alternately applied. This is a complex issue, the result of which may vary depending on the geometry of interest. As a result, there is uncertainty about how to proceed in order to discern whether a feature should be manufactured by either subtractive or additive means and thus optimize the sequence of operations. This task requires an in-depth knowledge of both technologies, as well as the consideration of part inspection as a built-in functionality able to update the manufacturing strategies in the process. This might be the last step to be accomplished in order to fully utilize hybrid machines.
- The inspection of additively manufactured parts is another challenging field of research in which advancements are being made. This is because such parts present a level of complexity in geometry that is unprecedented and metrology tools and measuring procedures need to be developed or adapted to them.
- Moreover, and despite the fact that machine tool mechatronics and process integration is nearly solved, there are still key aspects that need to be improved, such as programming tools, process monitoring and powder recovery.

In spite of overcoming the technological challenges mentioned above, the mindset of designers and engineers must be changed in order to fully understand and utilize additive manufacturing technologies. This way, a more efficient use of machines, materials, and resources might be attained.
